# Fecal carriage and clonal dissemination of *bla*_NDM-1_ carrying *Klebsiella pneumoniae* sequence type 147 at an intensive care unit in Lao PDR

**DOI:** 10.1371/journal.pone.0274419

**Published:** 2022-10-04

**Authors:** Tsegaye Sewunet, Sriram K. K., Ha Hoang Nguyen, Noikaseumsy Sithivong, Ngoc Thi Bich Hoang, Vanphanom Sychareun, Kokasia Nengmongvang, Mattias Larsson, Linus Olson, Fredrik Westerlund, Christian G. Giske

**Affiliations:** 1 Division of Clinical Microbiology, Department of Laboratory Medicine, Karolinska Institutet, Stockholm, Sweden; 2 Department of Biology and Biological Engineering, Chalmers University of Technology, Gothenburg, Sweden; 3 Hanoi Medical University, Hanoi, Vietnam; 4 Training and Research Academic Collaboration (TRAC) Sweden, Vietnam; 5 National Center for Laboratory and Epidemiology, Ministry of Health, Vientiane, Lao PDR; 6 Department of Microbiology, Vietnam National Children’s Hospital, Hanoi, Vietnam; 7 Faculty of Postgraduate Studies, University of Health Sciences, Ministry of Health, Vientiane, Lao PDR; 8 Department of Global Public Health, Karolinska Institutet, Stockholm, Sweden; 9 Department of Women’s and Children’s Health, Karolinska Institutet, Stockholm, Sweden; 10 Clinical Microbiology, Karolinska University Hospital, Stockholm, Sweden; Ross University School of Veterinary Medicine, SAINT KITTS AND NEVIS

## Abstract

**Objectives:**

Carbapenemase-producing *Enterobacterales* (CPE) are high priority targets of global antimicrobial surveillance. Herein, we determined the colonization rate of CPE on admission to intensive care units in Vientiane, Lao PDR in August-September 2019.

**Methods:**

Data regarding clinical conditions, infection control, and antibiotic usage were collected during admission. Rectal swab samples (n = 137) collected during admission were inoculated to selective chromogenic agars, followed by confirmatory tests for extended-spectrum beta-lactamases and carbapenemases. All CPE isolates were sequenced on Illumina (HiSeq2500), reads assembled using SPAdes 3.13, and the draft genomes used to query a database (https://www.genomicepidemiology.org) for resistome, plasmid replicons, and sequence types (ST). Optical DNA mapping (ODM) was used to characterize plasmids and to determine location of resistance genes. Minimum spanning tree was generated using the Bacterial Isolate Genome Sequence database (BIGSdb) and annotated using iTOL.

**Result:**

From 47 *Enterobacterales* isolated on selective agars, *K*. *pneumoniae* (25/47) and *E*. *coli* (12/47) were the most prevalent species, followed by *K aerogenes* (2/47), *K*. *variicola* (1/47), and *K*. *oxytoca* (1/47). The overall prevalence of ESBLs was 51.0%; *E*. *coli* 83.3% (10/12) and *Klebsiella* spp. 41.3% (12/29). Twenty percent of the *K*. *pneumoniae* (5/25) isolates were carbapenem-resistant, and 4/5 contained the *bla*_NDM-1_ gene. All *bla*_NDM-1_ isolates belonged to ST147 and were indistinguishable with cgMLST. ODM showed that the *bla*_NDM-1_ gene was located on identical plasmids in all isolates.

**Conclusion:**

The prevalence of ESBL-producing *Enterobacterales* was high, while carbapenemases were less common. However, the detection of clonal dissemination of *bla*_NDM-1_-producing *K*. *pneumoniae* isolates in one of the intensive care units calls for vigilance. Stringent infection prevention and antimicrobial stewardship strategies are highly important measures.

## 1. Introduction

Antimicrobial resistance is one of the major crises in human and animal health [1]. In a recent systematic analysis of globally available data, antimicrobial resistance was estimated to cause huge loss of economy and life in the coming decades [2]. Resistance to the most clinically important antimicrobial classes, third-generation cephalosporins and carbapenems, is a global problem [3].

Enzymatic hydrolysis of the β-lactam ring of third generation cephalosporins by extended spectrum-β-lactamases (ESBLs) is one of the main mechanisms of resistance. The *bla*_CTX-M-15_ gene is widely spread, and is the predominant ESBL-gene encoded by most epidemic isolates [3]. Similarly, in carbapenemase-producing *Enterobacterales* (CPE) the most important mechanism of resistance for epidemiological purposes is the production of carbapenemases. The *bla*_NDM_, *bla*_KPC_, *bla*_VIM_, *bla*_IPM_ and *bla*_OXA-48_-group are the most common carbapenemase-encoding genes–mostly located on plasmids [4–7]. The prevalence, and distribution of carbapenemase-encoding genes varies considerably in different parts of the world. In Southeast Asia, *bla*_NDM_ has been reported as the most prevalent carbapenemase in India, Thailand, and Vietnam [8,9].

In a previous survey conducted at six hospitals in Lao PDR, the overall antibiotic usage was 71% and varied between hospitals from 50% to 88%. The most commonly prescribed antibiotic was ceftriaxone (40%), and the most common inappropriate use was for surgical prophylaxis (99%) [10]. Meanwhile, the Lao PDR has developed a national action plan for tackling antimicrobial resistance and inappropriate use of antimicrobials. The strategic plan aims to reduce the use of antimicrobials by 20% and healthcare-associated infections by 30%, by 2023 (https://www.who.int/publications/m/item/lao-pdr-national-strategic-plan-on-antimicrobial-resistance-in-lao-pdr-2019-2023). For the strategic plan to meet the set goals, knowledge of local epidemiology is important. Data regarding resistant strains, spread of high-risk clones and mechanism of resistance, including epidemiologically important plasmids, are critical to curb the spread of antimicrobial resistance. Moreover, cost effective and innovative diagnostic technologies that can be used at the site with less technical complexity is equally desired. Generally, it is important to fully understand the magnitude and dynamic of antimicrobial resistance, practice of antibiotic selection, implementation of infection prevention, and antimicrobial stewardship programs in Lao PDR. In this context, the intensive care units are considered hot spots for emergence of antimicrobial resistance and are therefore well suited for sentinel surveillance.

In this study, we determined colonization rate of ESBL-E and CRE among patients admitted to intensive care units of five selected hospitals in Lao PDR. Then, we characterized molecular features of carbapenemase-producing isolates and the plasmids carrying the gene.

## 2. Materials and methods

### 2.1 Patient characteristics

Patients admitted to intensive care units at five selected hospitals in Laos PDR were enrolled in the study from August to September 2019. The study was ethically approved by the Ministry of Health University of Health Sciences Ethical Committee, Lao PDR (recordal 062–18). A total of 137 rectal swab samples, a single swab from each patient, were collected from all patients admitted to the intensive care units. Clinical and demographic data and were collected by using structured electronic case report forms and entered into Google Forms.

### 2.2 Study site

The study was conducted in five central hospitals in Laos: Mittaphab, Mahosot, Sethathirath, Women and Children’s Hospital, and Children Hospital. Mittaphab hospital, specialized in traumatology, has 538 beds, and had an average of 300 bed occupancy per day. Mahosot is a 630 beds hospital with 400 bed occupancy per day. Sethathirath hospital, specialized in endocrines, cancer, and HIV/AIDS, has 250 beds, with full occupancy per day. The women’s and children’s hospital has 150 beds with full occupancy per day. The children hospital has 70 beds with 100 patients per day (https://data.humdata.org/dataset/who-data-for-lao-people-s-democratic-republic, https://www.healthdata.org/laos).

### 2.3 Isolation, identification and characterization of bacterial isolates

Rectal swabs were inoculated to chromogenic agar for *Enterobacterales* (CHROMID^®^ESBL, bioMerieux). Pink (*E*. *coli*) and blue (*Klebsiella spp*., *Enterobacter spp*) colonies were isolated and species identification was done by matrix assisted laser desorption ionization-time of flight (MALDI-TOF) mass spectrometery at the clinical microbiology laboratory, Karolinska University Hopsital. ESBL-phenotyping was performed primarily by screening using CHROMID^®^ESBL agar, followed by combined disk test, a confirmatory test using cefotaxime (Oxoid) and ceftazidime (Oxoid) with and without clavulanic acid (Oxoid).

Antimicrobial susceptibility testing was performed using disk-diffusion method, (https://www.eucast.org/ast_of_bacteria/disk_diffusion_methodology/).

Genomic DNA was extracted using EZ1^®^DNA Tissue Kit (QIAGEN) by using the EZ1 Advanced DNA Bacteria Card on EZ1 Advanced extraction system. NEXTRA-XT kits were used for library preparation and sequencing was performed on Illumina (HiSeq2500) platform at *Science for Life Laboratories*, Stockholm.

Raw reads were assembled using SPAdes 3.13., and the assembled draft genomes were used to query database like https://cge.cbs.dtu.dk/services/ResFinder/ for resistome profile of the isolates, https://cge.cbs.dtu.dk/services/PlasmidFinder/ for plasmid replicon typing, https://bigsdb.pasteur.fr/klebsiella/klebsiella.html for multilocus sequence typing and https://kaptive-web.erc.monash.edu/ for capasular and O-ligolippopolysaccharides serotype predictions.

### 2.4 Optical DNA mapping of plasmids

Plasmids were extracted using MACHERY-NAGEL kits, and characterized by optical DNA mapping (ODM) as described previously [11,12]. Briefly, the extracted plamsids were mixed with Cas9 protein (600 ng, Sigma Aldrich) in the presence of equimolar amounts of crRNA (5’CCGCTGCATTGATGCTGAGC3’ for the *bla*_NDM-1_ gene, and 5’ CCGTCGCGATGTATTAGCGT 3’ for the *bla*_CTX-M-15_ gene) and tracrRNA (0.5 nmol each, Dharmacon Inc.) in 1X NEB-3 buffer (New England Biolabs) and 1X bovine serum albumin (BSA, 0.1 μg/ml) and incubated at 4°C for 30 min. Post Cas9-cutting, the samples were subjected to a one-step competitive binding labeling at 50°C for 30 min in presence of YOYO-1 (1:2 bp molar ratio, Invitrogen) and Netropsin (60:1 bp molar ratio, Sigma-Aldrich) [13]. λ-DNA (48,502 bp, New England Biolabs) was added as an internal size reference [14]. ODM was performed using nanofluidic devices consisting of 120 parallel nanochannels of dimensions 100 nm (height), 150 nm (width) and 500 μm (length), the fabrication details of the nanofluidic devices is discussed in detail elsewhere [15]. Images were collected using an inverted fluorescence microscope (Zeiss AxioObserver.Z1) with 100X oil objective (Zeiss, 1.46 N.A.), FITC filter (488 nm excitation and 530/50 nm emission) and a Photometrix Evolve sCMOS camera. Image processing was performed using custom written Matlab codes as discussed previously [11,16]. Briefly, kymographs were generated from the images, processed to generate barcodes, intensity patterns along the DNA originating from the variation in YOYO-1: Netropsin binding at GC and AT rich regions, respectively. Barcodes from different experiments were compared against each other to identify any similarity between them. If p < 0.01, the barcodes were considered similar and if p > 0.01, the barcodes were considered not similar.

### 2.5 Ethical approval

Ethical clearance was obtained from Ministry of Health, University of Health Sciences Ethics Comittee, Loa PDR (recordal 062–18). Informed, and written consent was obtained from each study subjects, for minors consent was obtained from either parents or legal guardians.

## 3. Results

### 3.1. Clinical and demographic data

Overall, 137 patients admitted for more than 48 hours in five selected hospitals, Mittaphab Hospital (34, 24.8%), Mahosot Hospital (36, 26.9%), Women’s and Children’s Hospital (35, 25.6%), Sethathirath Hospital (6, 4.3%), and Children’s Hospital (26, 19.0%), were enrolled in the study. Most of the study participants were from neonatal (37.2%) and adult (36.0%) intensive care units. Among the 61 infants, 30 (49.2%) were full-term, and 31 (50.8%) were pre-term. The most common mode of delivery was vaginal delivery (77.1%) (**Table 1**).

**Table 1:** Demographic and clinical characteristics of the study participants.

		Participants	
		N	%
**Hospital (n = 137)**			
	Mittaphab Hospital	34	24.8
	Mahosot Hospital	36	26.3
	Women and Children’s Hospital	35	25.5
	Sethathirath hospital	6	4.4
	Children hospital	26	19.0
**Department (n = 137)**			
	Neonatal ICU	51	37.2
	Pediatric ICU	30	21.9
	General ICU	6	4.4
	Adult ICU	50	36.5
**CRE screening (n = 137)**			
	Screening conducted < 48 hours after admission	28	20.4
	Screening conducted > 48 hours after admission	109	79.6
**Sex (n = 137)**			
	Female	59	43.1
	Male	78	56.9
**Age (n = 137)**			
	Up to 1 month old	62	45.3
	1m -14 years old	19	13.2
	15–64 years old	40	29.2
	65 years old	16	11.7
**Infant Gestational age (n = 61)**			
	Full-term	30	49.2
	Pre-term	31	50.8
**Infant Delivery (n = 61)**			
	Vaginal	47	77.1
	Caesarean section	8	13.1
	Emergency cesarean	6	9.8

A majority of the patients (73.7%) had not taken any antibiotic within three months before admission to the hospitals, but when used the most commonly used were oral and interavenous cephalosporins and penicillins (S1 Fig). The most commonly prescribed antibiotics after admission to the intensive care units were cephalosporin followed by penicillins, and carbapenems (S2 Fig). The most commonly used infection control measures were gloves (n = 131), mask (n = 129) and hand desinfection (n = 131) in the admission ward (S3 Fig).

### 3.2 Bacterial isolates and antibiotic susceptibility testing

**Fig 1:**
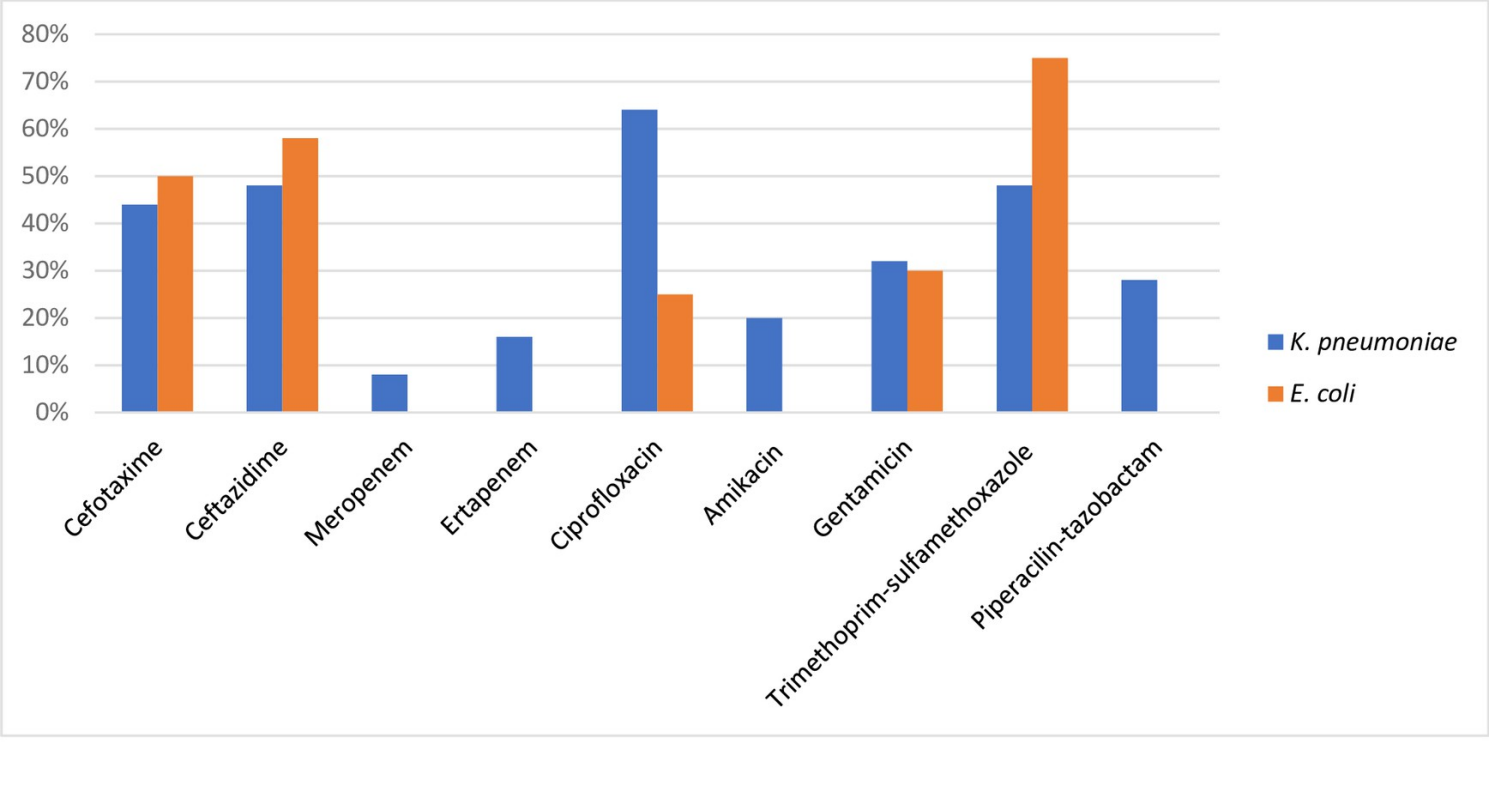
Antimicrobial susceptibility pattern for *E*. *coli* (n = 12*)* and *K*. *pneumoniae* (25) isolates on selective ESBL Chromagar and tested against commonly prescribed antibiotics.

A total of 47 bacteria belonging to *Enterobacterales* were isolated. *K*. *pneumoniae* (53.1%), and *E*. *coli* (25.5%) were the predominant species, followed by *E*. *cloacae* (n = 3), *K*. *aerogenes* (n = 2), *K*. *oxytoca* (n = 1), *K*. *variicola* (n = 1), and *S*. *marcescens* (n = 3). The proportion of ESBL-producing bacteria was high among both *E*. *coli* (10/12, 83.3%), and *Klebsiella* spp. (12/29, 41.3%). Some of the strains were resistant to multiple classes of antibiotics commonly prescribed in these hospitals, such as cefotaxime, ceftazidime, meropenem, ertapenem, ciprofloxacin, amikacin, and trimethoprim-sulfamethoxazole (Fig 1).

Five (20%) of the *K*. *pneumoniae* isolates were resistant to meropenem and four of them which encoded the *bla*_NDM-1_ gene were detected at one hospital. All the *bla*_NDM-1_ encoding isolates additionally encoded multiple resistance genes, including *bla*_CTXM-15._ Furthermore, all the carbapenemase-producing isolates encoded several resistance genes for different classes of antibiotics (aminoglycosides, extended-spectrum cephalosporins, fluoroquinoles, phenicoles, sulphonamides, and trimethoprim-sulfamethoxazole, S1 Table).

### 3.3 Core genome analysis (cgMLST) and plasmid replicon typing

**Fig 2:**
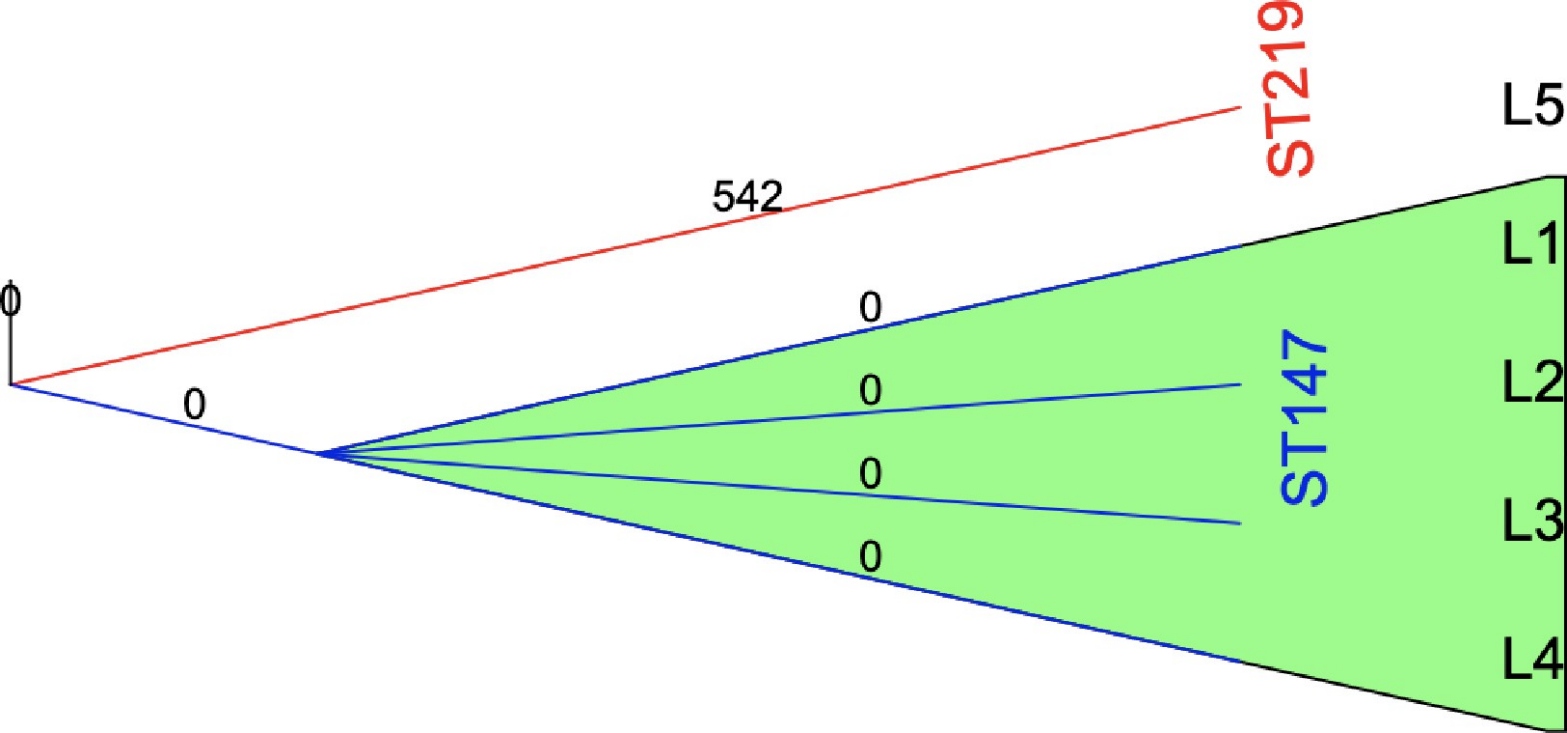
Minimum spanning tree constructed based on the dynamic core genome (cgMLST) of the *K*. *pneumoniae* isolates. The *bla*_NDM-1_ encoding ST147 isolates were clonally clustered (L1-L4, green shaded) and no allelic differences were detected.

Plasmid replicon typing showed the presence of three plasmids (*IncFIB(K)*, *IncFIB(*pQil) and *IncR*) in the *bla*_NDM-1_ encoding isolates and these isolates also encoded KL64 capsular type and the O2v1 O-LPS. The type of plasmid replicon, capsular locus, and O-LPS showed that the four carbapenemase-producing strains (L1—L4) encoded similar profiles (S2 Table). A cgMLST analysis showed that they belong to ST147 and that they had identical cgMLST profiles. L5 (ST219) was unrelated to the other four isolates and was not found to encode any carbapenemase genes (Fig 2).

### 3.4 Location of the *bla*_CTX-M-15_ and *bla*_NDM-1_ on identical plasmids

**Fig 3:**
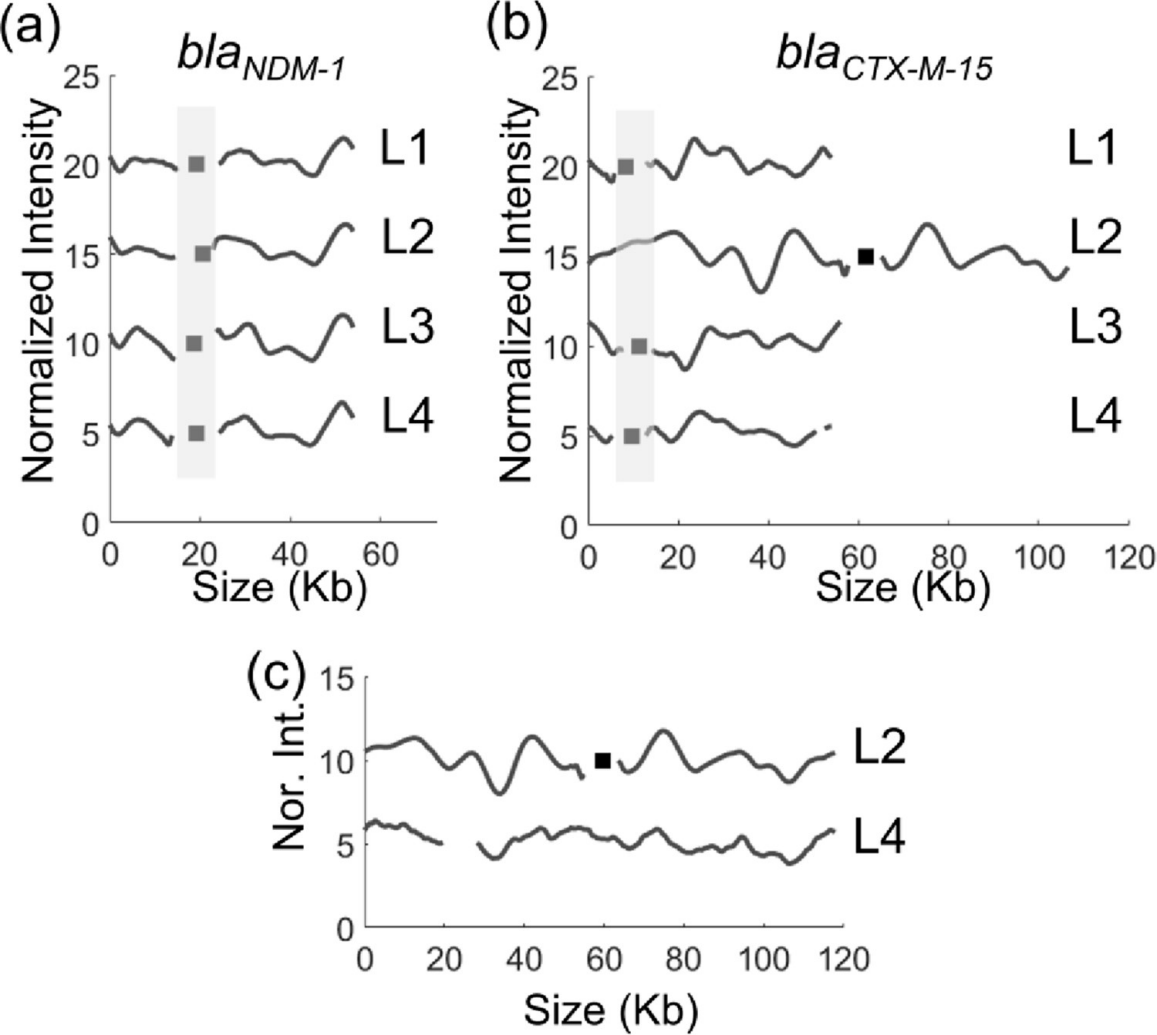
The barcode and gene location of the *bla*_NDM-1_ gene (a) and the *bla*_CTX-M-15_ gene (b) on plasmids isolated from the four carbapenemase-producing isolates L1-L4. (c) ~118 kb plasmid identified in isolates L2 and L4, the plasmid in L2 had the *bla*_CTX-M-15_ gene while L4 had neither the *bla*_NDM-1_ nor the *bla*_CTX-M-15_ gene. Black squares represent the gene location. The x-axis shows the size of plasmids in kb and the y-axis is the normalized intensity in arbitrary units, where the barcode of each isolate is shifted vertically by five units for clarity.

We have recently demonstrated that plasmid typing using ODM can be a useful method to trace clonal bacterial spread [17]. ODM is a method that in a single experiment yields the number of different plasmids in a sample, their size and on which plasmid a specific (resistance) gene is located, by forming a “barcode” along the DNA [16,18]. From the ST147 strains encoding the *bla*_CTX-15_ and *bla*_NDM-1_ genes, plasmids were extracted and analyzed by ODM. The barcodes from the ODM experiments showed that the *bla*_NDM-1_ gene was present on a plasmid of length around 54 kb in all four isolates. Both the barcodes and gene locations were identical between all the four isolates (Fig 3A, p<0.01).

Next, we did the same experiment, but instead targeting the *bla*_CTX-M-15_ gene (Fig 3B**)**. For L1, L3 and L4 we observed that the *bla*_CTX-M-15_ gene was located on the same plasmid as the *bla*_NDM-1_ gene and that the two genes were separated by approximately 10 kb (Fig 3C). However, for L2 the *bla*_CTX-M-15_ gene was located on another plasmid with a size of 118 kb.

We further investigated if the 118 kb plasmid was present in any of the other three *bla*_NDM-1_ encoding isolates. We identified a similar plasmid of size ~110 kb (p < 0.01) in isolate L4, but not in L1 and L3. The 110 kb plasmid in L4 did not encode *bla*_NDM-1_ or *bla*_CTX-M-15_. Further, we identified a 174 kb plasmid in isolates L1, L3 and L4 that did not encode *bla*_NDM-1_ or *bla*_CTX-M-15_ (S4 Fig).

## 4. Discussion

Herein, we screened for gut colonization of ESBL-E and CRE among patients admitted to intensive care units at five selected hospitals in Lao PDR in August and September in 2019. A previous systematic review and meta-analysis has shown that patients admitted to the intensive care units are more likely to be colonized with ESBL-producing *Enterobacteriaceae*, and previous colonization was found to be risk factor of infection [19]. Hence, detection of carbapenem-resistant isolates at an intensive care unit severely compromises safety of patients admitted to the ward. Colonization rates for CRE in neighbouring countries are high [20–22], calling for vigilance also in Laos.

The colonization rate detected was 17.5% for ESBL-E and 3.6% for CRE. Carbapenem resistance and the *bla*_NDM-1_ genes were detected only in *K*. *pneumoniae*. All the CR-KP isolates were found to encode multiple resistance genes, including *bla*_NDM-1_ and *bla*_CTX-M-15_. In addition, these isolates were highly similar in all typing methods performed. Most importantly, all the four *bla*_NDM-1_ encoding isolates were indistinguishable based on cgMLST phylogenetic analysis, suggesting that there was a small clonal outbreak in the hospital.

Moreover, we characterized plasmids from the CR-KP isolates with ODM, which demonstrated that the *bla*_NDM-1_ gene was located at same location on identical plasmids in all isolates. Interestingly, for three out of four isolates, the *bla*_CTXM-15_ gene was located on the same plasmid as the *bla*_NDM-1_ gene, indicating that the two genes were co-transmitted. The potential use of ODM of plasmids to identify clonal spread of bacteria in hospital settingss has been discussed before in studies at a tertiary hospital in Ethiopia [11], and at a neonatal intensive care unit in Sweden [23]. The results obtained here confirm the potential use of ODM for this purpose. Emergence of multidrug-resistant isolates with the potential to spread through either expansion of the high-risk clone as ST147, or via epidemic plasmids by horizontal genetic transfer, is challenging. The general situation of carbapenemases in Loa PDR is unknown, however, neighboring regions like Thailand, Vietnam, and southern parts China have reported higher prevalence of carbapenemases. Although carbapenems are not yet on the national list of essential drugs in Laos, the high prevalence of ESBL-producing *Enterobacterales* in Vientiane has driven widespread use of carbapenems imported by individual pharmacies from neighbouring countries. One previous study reported the *bla*_NDM-1_ gene from *E*. *coli* and *A*. *baumannii* [24], and the prevalence carbapenemases might be on the rise despite the lack of sufficient evidence. In 2017, infection prevention, and control strategies were nationally adopted in Lao PDR, and instituted at intensive care units in these hospitals. However, the *bla*_NDM-1_ colonization was detected from patients admitted to a special area in the ward, where observation of adherence to infection control measures was routinely performed. These patients were mainly admitted to the hospital for non-communicable diseseases like stroke, hemorrhage, and traumatic brain injury, at the intensive care unit in the same hospital. On the other hand, antibiotic usage was high after admission to the intensive care units. In the presence of high colonization rate of ESBL-E circulating, and the *bla*_NDM-1_ gene emerging at these hospitals, strict regulation of prescription, and routine antimicrobial susceptibility testing is important. Moreover, evaluating and strengthening of the current infection prevention and control strategy is necessary.

## 5. Conclusions

The emergence of ESBL-E and CRE isolates at an intensive care unit in low-income countries like Lao PDR is concerning. Moreover, clonal dissemination of the high-risk *K*. *pneumoniae* clone carrying an epidemic, conjugative plasmid, which in turn encodes mutiple resistance genes is a potential severe burden for a low-income country like Lao PDR. Systematic surveillance, strict prescription policy, and re-defining the infection prevention protocols are important strategies to curb the spread of these strains. Characterization of the epidemic plasmids using ODM can support in strengthening the infection prevention and control strategies in low-income settings.

## Supporting information

S1 Fig Antibiotic consumption of the study participants before admission to the intensive care units. (DOCX)

S2 Fig Types of and number of antibiotic prescriptions for study participants after admission to the intensive care units. (DOCX)

S3 Fig Infection control measueres implemeted for patients during the study period. (DOCX)

S4 Fig ~174 kb plasmid identified in isolates L1, L3 and L4. The x-axis shows the size of plasmids in kb and the y-axis is the normalized intensity in arbitrary units, where the intensity plot of each isolate is shifted vertically by five units for clarity.
(DOCX)

S1 Table Resistome profile of phenotypically carbapenem non-susceptible *K*. (DOCX)

S2 Table Sequence types, capsular and O-lipopolysaccharide types, encoded siderophore, and plasmid replicon types among carbapenem non-susceptible *K*. *pneumoniae* isolates (n = 5). (DOCX)
