# Supplementary material for: Fecal carriage and clonal dissemination of blaNDM-1 carrying Klebsiella pneumoniae sequence type 147 at an intensive care unit in Lao PDR
Source: PLoS One. 2022 Oct 4;17(10):e0274419. doi: 10.1371/journal.pone.0274419 (PMC9531820; doi:10.1371/journal.pone.0274419)
Supplement: S3 Fig — (DOCX) [file pone.0274419.s003.docx]

**Supplementary Figure 3**: Infection control measueres implemeted for patients during the study period.
