# Supplementary material for: Fecal carriage and clonal dissemination of blaNDM-1 carrying Klebsiella pneumoniae sequence type 147 at an intensive care unit in Lao PDR
Source: PLoS One. 2022 Oct 4;17(10):e0274419. doi: 10.1371/journal.pone.0274419 (PMC9531820; doi:10.1371/journal.pone.0274419)
Supplement: S4 Fig — The x-axis shows the size of plasmids in kb and the y-axis is the normalized intensity in arbitrary units, where the intensity plot of each isolate is shifted vertically by five units for clarity. (DOCX) [file pone.0274419.s004.docx]

**
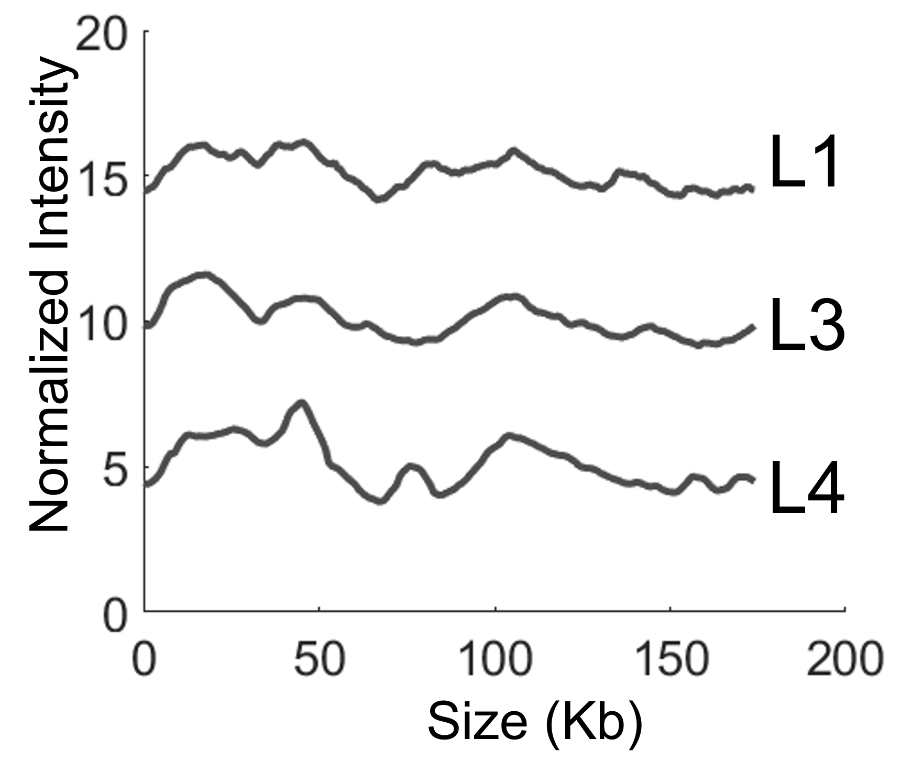
**

**Supplementary figure 4:** ~174 kb plasmid identified in isolates L1, L3 and L4. The x-axis shows the size of plasmids in kb and the y-axis is the normalized intensity in arbitrary units, where the intensity plot of each isolate is shifted vertically by five units for clarity
