# Supplementary material for: Fecal carriage and clonal dissemination of blaNDM-1 carrying Klebsiella pneumoniae sequence type 147 at an intensive care unit in Lao PDR
Source: PLoS One. 2022 Oct 4;17(10):e0274419. doi: 10.1371/journal.pone.0274419 (PMC9531820; doi:10.1371/journal.pone.0274419)
Supplement: S1 Table — (DOCX) [file pone.0274419.s005.docx]

**Supplementary table 1:** Resistome profile of phenotypically carbapenem non-susceptible *K. pneumoniae* isolates (n=5)

| Strains | *aadA1* | *aadA2* | *aph (3’’) Ib* | *aph (3’) Ia* | *aph (3^’^) -VI* | *aph (6’) Id* | *bla_CTX-M-15_* | *bla_NDM-1_* | *bla_OXA-1_* | *bla_OXA-9_* | *bla_SHV-11_* | *bla-_SHV-199_* | *bla_TEM-1A_* | *aac (6’) -Ib-cr* | *fosA* | *mph(A)* | *catB3* | *oqxA/oqxB* | *qnrS1* | *ARR-3* | *sul1/sul2* | *tet(A)* | *dfrA12* |
| --- | --- | --- | --- | --- | --- | --- | --- | --- | --- | --- | --- | --- | --- | --- | --- | --- | --- | --- | --- | --- | --- | --- | --- |
| L1 | + | + | - | - | + | - | + | + | + | + | + | - | + | - | + | + | + | + | + | + | + | + | + |
| L2 | + | + | - | - | + | + | + | + | + | + | + | - | - | - | + | + | + | + | + | + | + | + | + |
| L3 | + | + | - | - | + | - | + | + | + | + | + | - | + | + | + | + | + | + | + | + | + | + | + |
| L4 | + | + | - | - | + | - | + | + | + | + | + | - | + | + | + | + | + | + | + | + | + | + | + |
| L5 | - | + | + | + | - | + | + | - | - | - | - | + | - | - | + | + | - | + | + | - | + | + | + |

**Key:** Presence of the gene (+), Absence of the gene (-)
