# Supplementary material for: Fecal carriage and clonal dissemination of blaNDM-1 carrying Klebsiella pneumoniae sequence type 147 at an intensive care unit in Lao PDR
Source: PLoS One. 2022 Oct 4;17(10):e0274419. doi: 10.1371/journal.pone.0274419 (PMC9531820; doi:10.1371/journal.pone.0274419)
Supplement: S2 Table — (DOCX) [file pone.0274419.s006.docx]

**Supplementary table 2:** Sequence types, capsular and O-lipopolysaccharide types, encoded siderophore, and plasmid replicon types among carbapenem non-susceptible *K. pneumoniae* isolates (n=5)

| Strain | Sequence type (ST) | KL-locus | O-LPS | Siderophores | Plasmid replicon |
| --- | --- | --- | --- | --- | --- |
| L1 | ST147 | KL64 | O2v1 | *ironN* | *IncFIB(K), IncFIB(pQil), IncR* |
| L2 | ST147 | KL64 | O2v1 | *ironN* | *IncFIB(K), IncFIB(pQil), IncR* |
| L3 | ST147 | KL64 | O2v1 | *ironN* | *IncFIB(K), IncFIB(pQil), IncR* |
| L4 | ST147 | KL64 | O2v1 | *ironN* | *IncFIB(K), IncFIB(pQil), IncR* |
| L5 | ST219 | KL114 | O1v1 | *------* | *IncFIB(K) pCAV1099-144)* |
